# Cervical lymph node metastasis from occult tonsillar squamous cell carcinoma with incidental ectopic parathyroid tissue: a case report

**DOI:** 10.1093/jscr/rjag306

**Published:** 2026-05-31

**Authors:** Paolo Izzo, Claudia De Intinis, Silvia Lai, Antonio D’Urso, Simone Sibio, Paolo Meloni, Pierfrancesco Di Cello, Luciano Izzo, Marcello Molle, Sara Izzo

**Affiliations:** Department of Surgery ``Pietro Valdoni'', Policlinico Umberto I, Sapienza University of Rome, Viale del Policlinico 155, 00185 Rome, Italy; Department of Surgery ``Pietro Valdoni'', Policlinico Umberto I, Sapienza University of Rome, Viale del Policlinico 155, 00185 Rome, Italy; Department of Translational and Precision Medicine, Nephrology Unit, Policlinico Umberto I, Sapienza University of Rome, Viale del Policlinico 155, 00185 Rome, Italy; Department of Surgery ``Pietro Valdoni'', Policlinico Umberto I, Sapienza University of Rome, Viale del Policlinico 155, 00185 Rome, Italy; Department of Surgery ``Pietro Valdoni'', Policlinico Umberto I, Sapienza University of Rome, Viale del Policlinico 155, 00185 Rome, Italy; Obstetrics and Gynecology Unit, Imperia Hospital, ASL1 Imperiese, Via Sant'Agata, 57, 18100 Imperia, Italy; UOC General Surgery Frosinone-Alatri at ASL Frosinone, Via Armando Fabi, 5, 03100 Frosinone, Italy; Department of Surgery ``Pietro Valdoni'', Policlinico Umberto I, Sapienza University of Rome, Viale del Policlinico 155, 00185 Rome, Italy; Multidisciplinary Department of Medical-Surgical and Dental Specialties, Plastic Surgery Unit, Università degli Studi della Campania “Luigi Vanvitelli”, Piazza Luigi Miraglia 2, 80138 Napoli, Italy; Multidisciplinary Department of Medical-Surgical and Dental Specialties, Plastic Surgery Unit, Università degli Studi della Campania “Luigi Vanvitelli”, Piazza Luigi Miraglia 2, 80138 Napoli, Italy

**Keywords:** ectopic parathyroid, tonsillar squamous cell carcinoma, metastasis, surgery

## Abstract

Parathyroid glands originate from the third and fourth branchial pouches, and alterations in their embryological migration may result in ectopic parathyroid tissue, a rare finding, particularly within cervical lymph nodes. We describe the case of a 74-year-old man presenting with neck swelling, weight loss, dysphagia, and respiratory symptoms. Clinical examination revealed a firm cervical mass, while ultrasound, fine needle aspiration, and computed tomography imaging were inconclusive. Surgical excision and histopathological evaluation revealed metastatic squamous cell carcinoma and incidental ectopic parathyroid tissue within a cervical lymph node. A tonsillar carcinoma was subsequently identified as the primary tumor, and the patient was treated with radio-chemotherapy. This case highlights the rarity of ectopic parathyroid tissue in lymph nodes, especially in the absence of calcium metabolism disorders, and emphasizes the importance of accurate differential diagnosis to avoid misinterpretation and inappropriate management.

## Background and introduction

Parathyroid glands are small endocrine glands located in the neck behind the thyroid, usually four in number. One pair of glands comes from the third brachial pocket and begins its migration during the seventh week of life, the other pair of parathyroids originates from the fourth brachial pocket and begins its descent a few weeks after those resulting from the third pocket. Abnormalities of this migration process are responsible for ectopias of the seat of the parathyroids [1–3].

The finding of parathyroid tissue away from the thyroid lodge is an uncommon event especially if this tissue is found in the context of the lymph node tissue [4–6].

We describe the clinical case of ectopic parathyroid tissue found in the context of a neck lymph node formation affected by secondary repetition of a squamous cell carcinoma of the tonsil.

## Case report

A 74-year-old man presented to the doctor with a laterocervical swelling that had been noticeable for about 2 months, accompanied by significant weight loss. The mass was covered by non-ulcerated, hyperemic skin and had a firm, elastic consistency. It was fixed to both superficial and deep planes and caused localized pain. The patient reported additional symptoms such as dysphagia (difficulty swallowing) and respiratory difficulties due to tracheal lateralization, though he did not have a fever, and his general health was otherwise normal.

The patient’s medical history included arterial hypertension, mild heart failure, alcoholism, and smoking, with no family history of cancer. Neurologically, he was within normal limits, without any deficits or concerning findings.

After consulting with an ear, nose, and throat specialist, the patient underwent a neck ultrasound that revealed a solid, well-vascularized mass with necrotic areas. The thyroid was mildly enlarged but otherwise homogeneous, with only minimal solid nodules present. The trachea was laterally displaced to the right, although there was no significant compression of the vascular or nerve bundles in the neck. No lymph node hyperplasia was noted in the anterior cervical region. Fine needle aspiration of the mass was performed, but the cytological examination was non-diagnostic.

**Figure 1:**
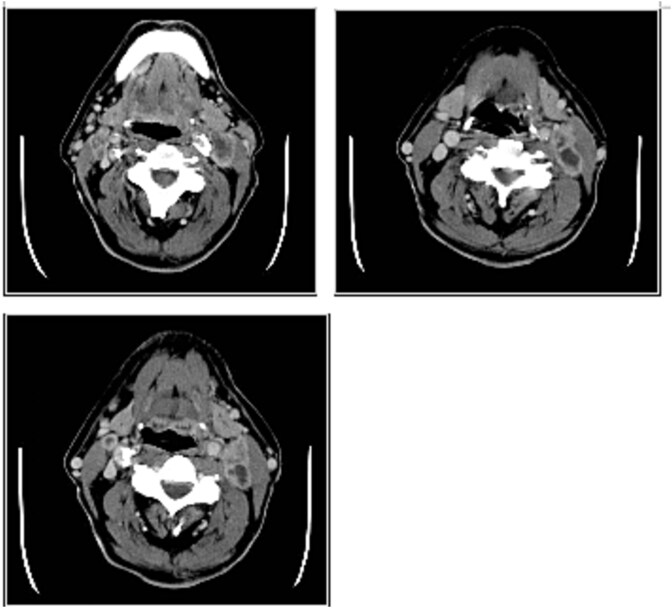
(a, b, c) Axial images in CT of the neck, where the latero-cervical mass is appreciated with impregnation of peripheral contrast medium, uneven with characteristics of malignancy and mimicking a latero-cervical lymphadenopathy.

Due to the non-conclusive results, a contrast-enhanced computed tomography (CT) scan of the neck was ordered to further investigate the origin of the suspected neoplasm. However, the scan did not reveal a clear primary tumor site (Fig. 1).

The patient underwent a selective laterocervical dissection, targeting the cervical lymph node stations of levels IIa, IIb, III, and IV. During the surgery, the removed tissue presented as a pinkish-gray mass measuring ~4 by 3 cm. The specimen was sent for pathological examination to the Institute of Pathological Anatomy for histological evaluation.

**Figure 2:**
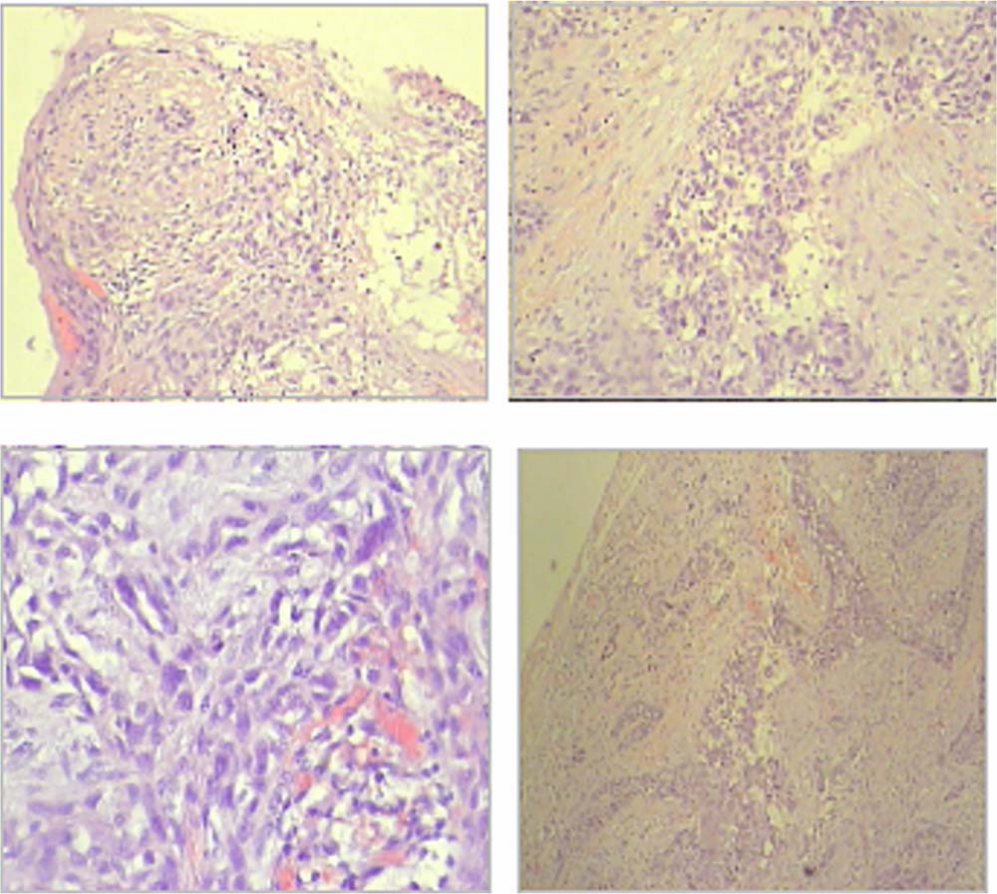
Microscopy—metastases from occult squamous cell carcinoma (hematoxylin and eosin staining); (a) overview, (b) dictation.

On macroscopic examination, a firm, grayish-yellow mass with necrotic areas was identified in a level III cervical lymph node. These characteristics raised suspicion of metastatic disease. Microscopic analysis confirmed the presence of metastases from an occult squamous cell carcinoma. Additionally, ectopic parathyroid tissue was unexpectedly found within the lymph node (Fig. 2).

During follow-up at 1, 3, and 6 months, a contrast-enhanced head and neck CT scan was performed, which revealed a suspicious area in the previously unidentified tonsillar region (tonsillar fossa). A biopsy of this area was carried out, and the histological examination confirmed the diagnosis of squamous cell carcinoma of the tonsil, thus identifying the primary tumor.

After multidisciplinary consultation, the patient was started on a course of radio-chemotherapy. The treatment plan consisted of a total of 35 cycles, aimed at addressing both the primary tumor and the metastatic disease. After completion of radio-chemotherapy, the patient underwent clinical and radiological follow-up up to 6 months, with no evidence of disease progression at the last evaluation. The patient subsequently did not attend further scheduled visits and was lost to follow-up.

## Discussion and conclusion

The peculiarity of our case lies in the identification of an ectopic parathyroid gland located at level III of the cervical lymph nodes in the absence of any alteration in calcium metabolism. In contrast, the literature mainly describes ectopic parathyroid glands associated with tumors that secrete hormones capable of altering calcium homeostasis, leading to metabolic imbalance and consequent bone resorption [5, 7–9].

The parathyroids derived from the third brachial pocket begin their migration during the seventh week of life, while the other pair, that originates from the fourth brachial pocket, begins its descent a few weeks later [9]. The finding of parathyroid tissue away from the thyroid lodge is an uncommon event especially if this tissue is found in the context of the lymph node tissue [10].

The clinical case studied by us showed a particular and rare position of parathyroid tissue, anatomically independent from the thyroid gland and far from the thyroid lodge and thymus, in the context of a lymph node formation, affected by secondary repetition. This tissue presented itself as an autonomous island from the surrounding lymphatic tissue, with its own vascularization. The infrequent finding of parathyroid parenchyma, totally independent and distant from the anterior region of the neck and the thyroid lodge, is reported in the literature with a percentage ranging from 4% to 10% according to the different cases reported [11–14]. Veras *et al*. highlighted that benign intranodal parathyroid inclusions may be encountered unexpectedly in neck dissection specimens and should be considered to prevent overdiagnosis of metastasis [15]. In all cases reported in the literature, however, squamous cell carcinoma tissue was not detected in these regions of ectopic parathyroid tissue.

One of the potential critical issues identified in our clinical case was the failure to perform a panendoscopy with targeted biopsies in order to identify an occult primary malignant neoplasm. In the case in question, the initial diagnostic process focused on imaging and cytological evaluation due to the patient’s clinical condition and the rapid progression of compressive symptoms, leading to surgical excision of the mass for diagnostic and therapeutic purposes. The primary tumor was subsequently identified during postoperative follow-up.

The ectopic parathyroid is to be diagnosed in a differential way with other pathologies that lead to an increase in the volume of lymph node stations and those that lead to alterations in the calcium-phosphorus balance after radical ablation of the thyroid gland. The need for differential diagnosis is of importance to avoid having to resort to a second surgery to remove the pathogen damage not initially recognized [16].
